# *In Silico* Investigation of Flavonoids as Potential Trypanosomal Nucleoside Hydrolase Inhibitors

**DOI:** 10.1155/2015/826047

**Published:** 2015-11-12

**Authors:** Christina Hung Hung Ha, Ayesha Fatima, Anand Gaurav

**Affiliations:** ^1^Faculty of Pharmaceutical Sciences, UCSI University, 1 Jalan Menara Gading, Taman Connaught, Cheras, 56000 Kuala Lumpur, Malaysia; ^2^Faculty of Medicine, Quest International University Perak, No. 227, Plaza Teh Teng Seng, Jalan Raja Permaisuri Bainun, 30250 Ipoh, Perak, Malaysia

## Abstract

Human African Trypanosomiasis is endemic to 37 countries of sub-Saharan Africa. It is caused by two related species of* Trypanosoma brucei*. Current therapies suffer from resistance and public accessibility of expensive medicines. Finding safer and effective therapies of natural origin is being extensively explored worldwide. Pentamidine is the only available therapy for inhibiting the P2 adenosine transporter involved in the purine salvage pathway of the trypanosomatids. The objective of the present study is to use computational studies for the investigation of the probable trypanocidal mechanism of flavonoids. Docking experiments were carried out on eight flavonoids of varying level of hydroxylation, namely, flavone, 5-hydroxyflavone, 7-hydroxyflavone, chrysin, apigenin, kaempferol, fisetin, and quercetin. Using AutoDock 4.2, these compounds were tested for their affinity towards inosine-adenosine-guanosine nucleoside hydrolase and the inosine-guanosine nucleoside hydrolase, the major enzymes of the purine salvage pathway. Our results showed that all of the eight tested flavonoids showed high affinities for both hydrolases (lowest free binding energy ranging from −10.23 to −7.14 kcal/mol). These compounds, especially the hydroxylated derivatives, could be further studied as potential inhibitors of the nucleoside hydrolases.

## 1. Introduction

Human African Trypanosomiasis (HAT), also known as sleeping sickness, is one of the 17 neglected tropical diseases listed by the WHO [1]. It is endemic in sub-Saharan Africa where the vector for its transmission, the tsetse fly (*Glossina* species), can be found. HAT is caused by two subspecies of* Trypanosoma brucei (T. b.)*, namely,* Trypanosoma brucei gambiense* and* Trypanosoma brucei rhodesiense* [2]. Following several control measures, the World Health Organization reported a drop in the new cases [3]. However, many more are believed to be unreported or undiagnosed especially those who live in remote areas.

*T. b. gambiense* is responsible for the chronic form of the disease that accounts for 98% of all reported cases while* T. b. rhodesiense* causes the acute disease accounting for 2% of the reported cases [1, 2]. The infection is divided into two stages for better therapeutic management. In the first stage, the parasite multiplies in the subcutaneous tissues after entering the host. This stage, which is also called the hemolytic phase, causes the first symptoms of headaches, fever, joint pains, and itching. In the second stage, namely, the neurological stage, the parasite crosses the blood-brain barrier and infects the central nervous system causing behavioral change, confusion, poor sensory coordination, and disturbances in the sleep cycle, all of which are the hallmarks of the disease. The prolonged asymptomatic first stage of the disease sometimes makes early diagnosis and treatment difficult.

Suramin is the first-stage drug for* rhodesiense*. The mode of action of suramin has been reported to be inhibition of glycerol-3-phosphate oxidase and dehydrogenase [4] as well as the inhibition of uptake of low-density lipoproteins and the consequent hampering of cell division [5]. Inhibitory effects of suramin on dihydrofolate reductase, L-a-glycerophosphate oxidase, dihydrofolate dehydrogenase, reverse transcriptase, thymidine kinase, trypsin, and RNA polymerase were also reported [6, 7]. Pentamidine is effective for treating first-stage* gambiense*. Its mode of action centers on the inhibition of P2 adenosine transporter in the parasites, hence inhibiting their replication. Melarsoprol is the only drug recommended for the second stage of both types of diseases. The primary target of melarsoprol was suggested to be the inhibition of trypanothione reductase, a key enzyme in detoxification processes in the trypanosomes [8]. However, Wang proposed inhibition of phosphofructokinase, a key enzyme in the glycolytic pathway [7]. Eflornithine (DFMO) has been successful only in the second stage of* gambiense* infection [9]. The drug is an inhibitor of ornithine decarboxylase (ODC), a key enzyme in polyamine biosynthesis [10]. All drugs are available only in the intravenous form. Nifurtimox, the only orally administered drug, acts by causing oxidative stress in the trypanosome. Combination therapy may be more effective than monotherapy for the treatment of late-stage* T. brucei gambiense* trypanosomiasis. The Nifurtimox-Eflornithine combination therapy was recently approved by the WHO for use in late-stage* T. brucei gambiense* trypanosomiasis [11, 12]. There is none available for late-stage* rhodesiense*. Most currently used drugs are toxic and resistance has been reported frequently [13].

**Figure 1:**
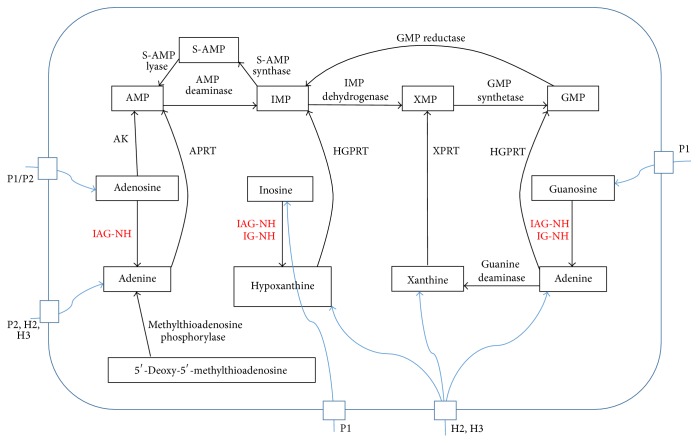
PSP of* T. brucei* showing key enzymes and transporters. AK = adenosine kinase, APRT = adenine phosphoribosyltransferase, HGPRT = hypoxanthine-guanine phosphoribosyltransferase, XPRT = xanthine phosphoribosyltransferase, and IG-NH = inosine-guanosine nucleoside hydrolase. P1, P2, P1/P2, H2, and H3 are purine base and/or nucleoside transporters [16].

Development of new safe and more efficacious drugs for HAT is an ongoing research because of the wide coverage area and easy transmission between humans and animal. Several studies on rational drug design report inhibition of key enzymes responsible for replication of the parasite. The purine metabolism pathway of* T. brucei* provides a valuable target in search of new selective antitrypanosomal drugs. In contrast to human hosts (mammals), all parasites are unable to synthesize purines* de novo* and solely rely on the purine salvage pathway (PSP) to satisfy their purine needs, which is essential for all stages of the parasite life cycle. As shown in Figure 1, the key enzymes in this pathway are the nucleoside hydrolases (NH). These enzymes catalyze the cleavage of the N-glycosidic bond of nucleosides to yield purine bases. The reaction can be simplified as follows:

(1) $$\begin{array}{c} \text{nucleoside}\overset{\text{NH}}{⟶}\text{purine base}+\text{pentose sugar} \end{array}$$

where the nucleoside can be inosine, guanosine, and/or adenosine depending on its specific preference of substrates. Two types of NH have been reported for* T. brucei*, the purine nucleoside specific inosine-adenosine-guanosine nucleoside hydrolase (IAG-NH) and the 6-oxopurine-specific inosine-guanosine nucleoside hydrolase (IG-NH) [14, 15]. Inhibition of NH depletes the level of purine bases in* T. brucei*, retarding their growth and multiplication. In addition, NH activity is absent in mammal purine biosynthesis pathways [16].

Berg et al. have shown significant trypanocidal effects of nucleoside hydrolase inhibitors without exhibiting cytotoxicity to human cell lines. This makes NH a good target for developing new cure for HAT [16]. Researchers also indicated that the NH inhibitors show isoenzyme selective inhibition towards IAG-NH and IG-NH due to the difference in active site features, and inhibition of either one enzyme alone is not sufficient to impair the PSP in the parasites [16].

**Figure 2:**
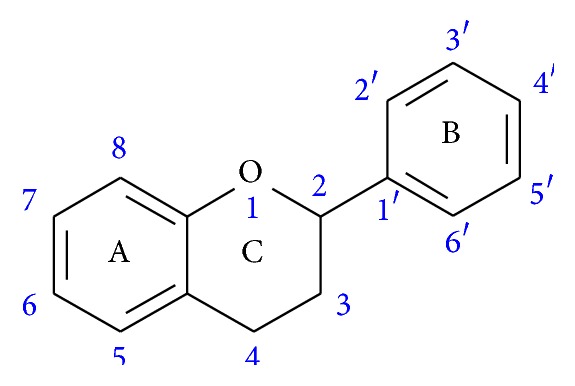
Basic flavonoid structure.

Flavonoids are polyphenolic compounds having a common benzo-*γ*-pyrone structure (Figure 2). They are present in many plants including those commonly found in human diet such as vegetables, fruits, grains, wine, and flowers. Flavonoids are well known for their diverse biological activities, particularly having antioxidant, anti-inflammatory, and antithrombogenic effects. Studies have also shown that flavonoids exert anticancer, antibacterial, and antivirus properties and can be safely used for therapeutic purposes without causing any visible toxic effects [17–22]. Excellent antitrypanosomal and antileishmanial activities of flavonoids have also been reported by various studies [23–25]. Among all compounds, eight flavonoids of various levels of hydroxylations, namely, flavone, 5-hydroxyflavone, 7-hydroxyflavone, chrysin, apigenin, kaempferol, fisetin, and quercetin, are selected for this study. Flavone is the nonhydroxylated compound while the hydroxyflavones, chrysin, and apigenin represent the mono-, di-, and trihydroxylated derivatives of flavone. On the other hand, kaempferol, fisetin, and quercetin belong to the flavan-3-ol subclass with kaempferol as the 3,5,7,4′-tetrahydroxylated derivative, quercetin having one additional hydroxylation at position 3′, and fisetin having all similar hydroxylations as quercetin except for the missing 5-OH.  Table 1 summarizes the test compounds and the respective positions of hydroxyl (-OH) groups.

*In silico* screening offers the advantage of identifying lead compounds from several potentially useful hits [26]. Molecular docking offers a very efficient and fast method to do so [27]. Researchers have employed the method successfully to determine potentially useful binding sites and used the results to identify, improve, and perhaps develop drugs that fit better into the binding pocket. Several simple pieces of software such as DOCK, AutoDock, and AutoDock Vina offer the advantage of locating the plausible pockets efficiently [28, 29].

Using docking technique, the study looks at the potential of flavonoids as inhibitors of nucleoside hydrolases which could be the probable mechanism of trypanocidal effect of the compounds. It is our interest to study the probable protein-ligand binding interaction and to draw structure-activity relationships.

## 2. Materials and Methods

The crystal structure of* T. b. brucei* IAG-NH (PDB ID: 4I71) resolved at 1.28 Å [15] and* T. b. brucei* IG-NH (PDB ID: 3FZ0) [30] were retrieved from the Protein Data Bank [31]. Both structures had cocrystallized ligands. Only chain A out of the four chains of 3FZ0 was used as a representative as all four chains are identical. The protein was processed for docking procedure using AutoDock Tools 1.5.6. All solvent molecules, water molecules, and the cocrystallized ligand and the allosteric inhibitor Ni^2+^ ion were removed from the structure; Kollman charges and polar hydrogens were added. The calcium ion in the enzymes was also removed in order to study the protein-ligand interactions without interference of the cation. The files were generated as PDBQT format. PDBQT file of the ligands was generated with all the default values accepted. Docking was performed by using AutoDock 4.2 [29] with grid spacing set at 2.0 Å and the grid points in *x*-, *y*-, and *z*-axis set to 36 × 36 × 36. The grid is centered at the cocrystallized ligands of the respective proteins. The search was done based on the Lamarckian genetic algorithm. The number of individuals in population is set to be 150, with a maximum of 2,500,000 energy evaluations and 27,000 generations. The rate of gene mutation is 0.02 while the rate of crossover is 0.8. For each ligand, the docking runs were set to be 50 and the analysis is done based on binding energies and Root Mean Square Deviation (RMSD) values. The ligands were ranked in ascending order of free binding energies.

**Figure 3:**
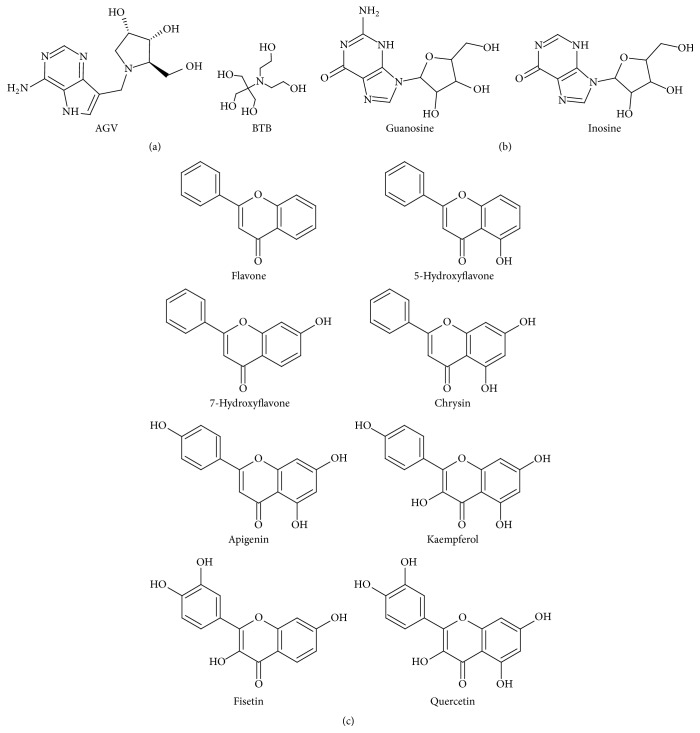
Structures of chemical compounds used in the study. (a) Chemical structures of AGV and BTB, the cocrystallized ligand of* T. b. brucei* IAG-NH and IG-NH, respectively. (b) Chemical structures of inosine and guanosine, the natural substrate of both* T. b. brucei* IAG-NH and IG-NH. (c) Chemical structures of the compounds tested: flavone, 5-hydroxyflavone, 7-hydroxyflavone, apigenin, fisetin, kaempferol, quercetin, and chrysin.

The 3D structures of the ligands are downloaded from PubChem. The selected binding sites for both enzymes were the ones reportedly occupied by the inhibitors AGV and BTB in the crystal structures [15, 30]. Controlled docking was performed with ligands AGV and BTB and two of the natural substrates, guanosine and inosine, for validation of the experimental protocol. All the test compounds were listed in Table 2, with the structures generated using ChemSketch [32] featured in Figure 3.

## 3. Results and Discussion

**Figure 4:**
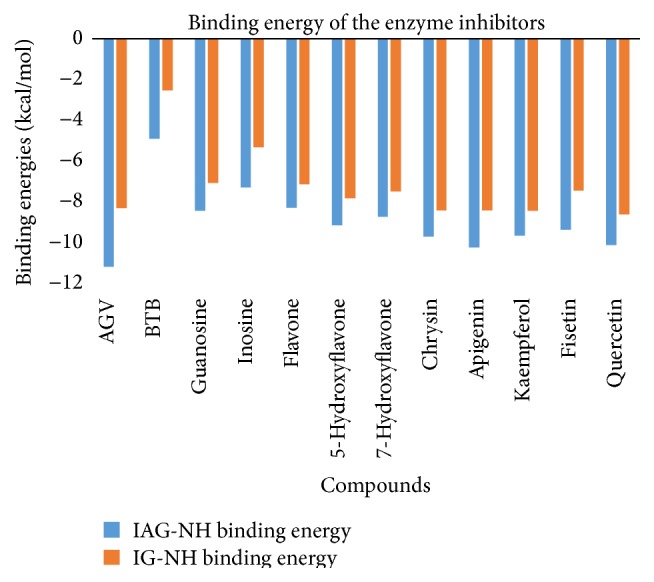
Docking results showing lowest free binding energy of compounds with IAG-NH and IG-NH from* T. b. brucei*.

**Figure 5:**
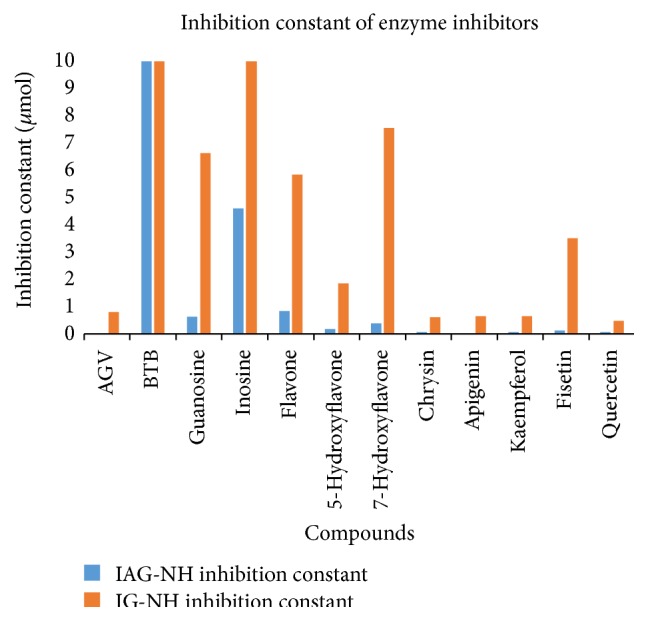
The estimated inhibition constant (Ki) from docking results of the compounds with IAG-NH and IG-NH from* T. b. brucei*.

For all ligands docked, the 50 runs were grouped into a range of 1-2 and 1–8 multimember conformational clusters for IAG-NH and IG-NH, respectively. All docking conformations were visualized using AutoDock Tools 1.5.6 so as to ensure the ligands were docked into the defined pocket.  Figure 4 presents the lowest binding energy from the largest cluster chosen to represent the result of each docking, regardless of the cluster rank. This is based on the rationale that conformations in the largest cluster are more statistically possible. The estimated inhibition constant, Ki, calculated at 298.15°K for the selected conformations was recorded as well. The results are presented in Figure 5.

**Figure 6:**
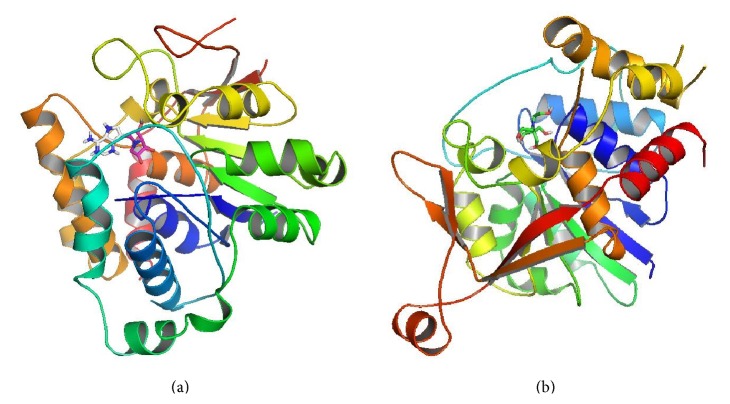
Docked poses of* T. b. brucei* NH with their cocrystallized ligands (stick figures). (a) IAG-NH with AGV; (b) IG-NH with BTB.

Figure 6 illustrates the docking poses of the controlled molecules. PDB files of the docked position were generated for control and best compounds using AutoDock Tools [29], which were then imported to LigPlot+ to generate two-dimensional ligand binding interaction plots and superimposed to compare the bindings of different ligands on the same protein and the same ligand on both NH [33]. Our results indicated that the binding pocket of IAG-NH was made of Asp10, Asn12, Lys13, Asp14, Asp15, Asp40, Phe79, Trp83, Thr137, Gly138, Met164, Gly165, Asn173, Glu184, Trp185, Asn186, Leu210, Glu248, Arg252, Asp255, Ala256, Tyr257, Trp260, and Asp261. As for IG-NH, the binding site residues were Asp11, Asp15, Asp16, Asn40, Trp80, Phe83, Leu131, Gly132, Met162, Asn171, Glu177, Phe178, Asn179, Trp205, Phe247, Leu250, Thr254, Thr275, Cys276, Val277, Val278, Pro279, and Asp280.

**Figure 7:**
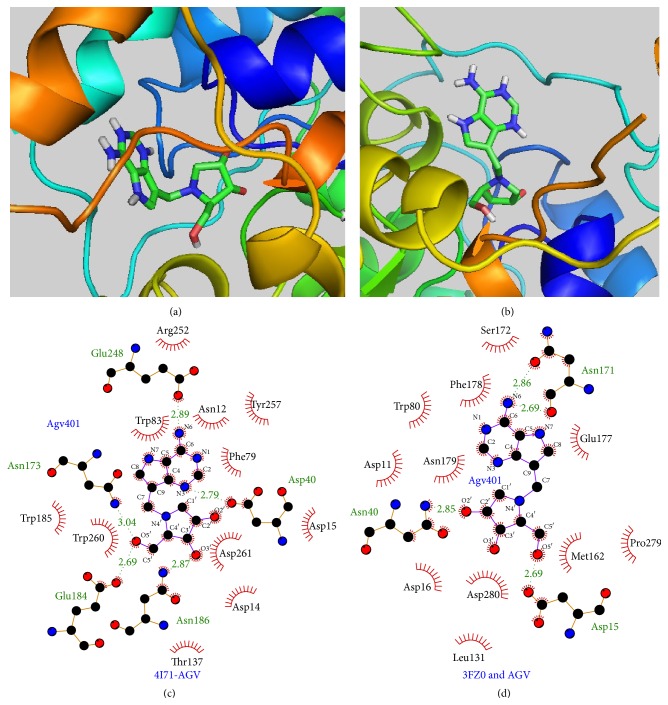
Docked poses of AGV with (a) IAG-NH and (b) IG-NH. LigPlot figures of AGV docked with (c) IAG-NH and (d) IG-NH.

Results also showed that AGV has the lowest free binding energy, −11.19 kcal/mol, against IAG-NH among all the tested compounds. The estimated Ki of AGV were 0.0063 *μ*M against IAG-NH and 0.8154 *μ*M against IG-NH. These results were in agreement with the experimental data by Berg et al. that showed the inhibition constant Ki of 0.018 *μ*M and 32 *μ*M against IAG-NH and IG-NH, respectively [16]. The binding energy of AGV towards IG-NH (−8.31 kcal/mol) is lower than IAG-NH (−11.19 kcal/mol) but considerably higher when compared to both natural substrates inosine and guanosine (binding energies −5.31 kcal/mol and −7.06 kcal/mol, resp.). This can be explained by the extensive hydrogen bonding of AGV in IAG-NH as compared to IG-NH, as shown in Figure 7.

The binding energy of BTB is comparatively lower towards both enzymes, −4.9 and −2.54 kcal/mol, with inhibition constant of >10 *μ*M. This is obvious from the structure of BTB which lacks the pyrrolidine and pyrrole rings required by the hydrophobic pocket to bind effectively as present in the natural substrates of the enzymes as well as AGV. The extensive OH groups bind to only four uncharged and negatively charged amino acids in the binding pocket thereby producing low binding energy and high binding constant (not shown).

All the eight test compounds showed considerably high selectivity (Figure 4) with estimated Ki at nanomolar concentration against IAG-NH enzyme. Tasdemir et al. [23] have done* in vitro* study on trypanocidal activity of these compounds along with several other classes of flavonoids. They reported that flavone, 5-hydroxyflavone, 7-hydroxyflavone, chrysin, apigenin, fisetin, kaempferol, and quercetin indicate IC_50_ of 6.4, 10.6, 6.6, 5.3, 5.1, 3.3, 9.2, and 8.3 micrograms/mL, respectively, against* T. b. rhodesiense* where melarsoprol, used as control, had IC_50_ values of 0.0026 micrograms/mL in the same experiment. Tasdemir et al. attributed the trypanocidal activity to the presence of hydroxyl groups on the flavone rings [23].

Our results also confirmed the observations of the authors, where strong hydrogen bonds were formed between the hydroxyl groups on the flavonoids with polar amino acid residues in the binding pocket of IAG-NH such as Asn173 and Glu184. For example, in case of flavone, that had the lowest energy (−8.28 kcal/mol) compared to other hydroxylated derivatives since it has no hydroxyl groups to offer. The carbonyl oxygen remains the main functional group to hydrogen bond with the residues in the binding pocket. Binding energy increases with each increase in number of hydroxyl groups from 0 in flavone (−8.28 kcal/mol) to 3 in apigenin (−10.23 kcal/mol). This phenomenon could also be seen in interaction of AGV and guanosine with the same protein. Quercetin with 5-hydroxyl groups had slightly less binding energy −10.12 kcal/mol. The reason for this change could be attributed to the position of the OH group discussed further below.

The position of -OH groups was also found to affect the binding energy. 5-OH position increases the binding energy of a flavone more than 7-OH. For example, flavone and 5-hydroxyflavone have binding energies −8.28 kcal/mol and −9.16 kcal/mol as compared with 7-hydroxyflavone that exhibits binding energy of −8.73 kcal/mol. We observed further that hydroxylation at position 5 significantly increases the binding energy, as seen in pairs of compounds: flavone and 5-hydroxyflavone: −8.28 kcal/mol and −9.16 kcal/mol; 7-hydroxyflavone and chrysin (5-OH and 7-OH): −8.73 kcal/mol and −9.40 kcal/mol; and fisetin (no 5-OH) and quercetin (-OH at 3, 5, 7, 3′, and 4′ positions): −9.37 kcal/mol and −10.12 kcal/mol.

Hydroxylation at positions 3 and 3′ was also found to be unfavorable. It can be seen that binding energy of apigenin is −10.23 kcal/mol with no hydroxyl groups at both stated positions while kaempferol has hydroxyl group at position 3 and its binding energy decreases to −9.67 kcal/mol. The binding energy of fisetin that has a hydroxyl group at 3′ in addition to position 3 is further decreased to −9.37 kcal/mol. Our results also indicated that hydroxylation at 4′ position could be responsible for improved binding energy such as in case of kaempferol and quercetin with binding energies of −9.67 kcal/mol and −10.12 kcal/mol. Addition of hydroxyl to positions 3 and 3′ decreased the binding energy such as the case of quercetin but the presence of hydroxyl group at positions 5, 7, and 4′ led to its comparatively better binding energy. Summarizing our results for IAG-NH, flavonoids with three hydroxyl groups at positions 5, 7, and 4′, respectively, as seen in apigenin seemed to be the most ideal combination.

In case of IG-NH, all compounds showed generally lower binding energies. Analysing the effect of hydroxylation pattern of flavonoids on the binding energies for IG-NH, flavone with no hydroxyl group had the lowest binding energy (−7.14 kcal/mol) among the flavonoids. Increasing the number of hydroxyl groups improved binding energies. Hydroxylation at positions 5 and 4′ remained important while position 3′ led to reduction of binding energy. The strongest bound molecule was quercetin with −8.61 kcal/mol binding energy.

Besides, the flavonoids also shared a similar pattern of extensive hydrophobic interactions with the protein residues. It can be proposed that the sum of both hydrogen bond and hydrophobic interactions contributed to the high affinity of these ligands towards the trypanosomal NH. Tasdemir et al. [23] had reported trypanocidal activity of the flavonoids against the whole organism. Combining our docking and their experimental results, it can be said that inhibition of nucleoside hydrolases could be the probable target of the trypanocidal activity of flavonoids.

**Figure 8:**
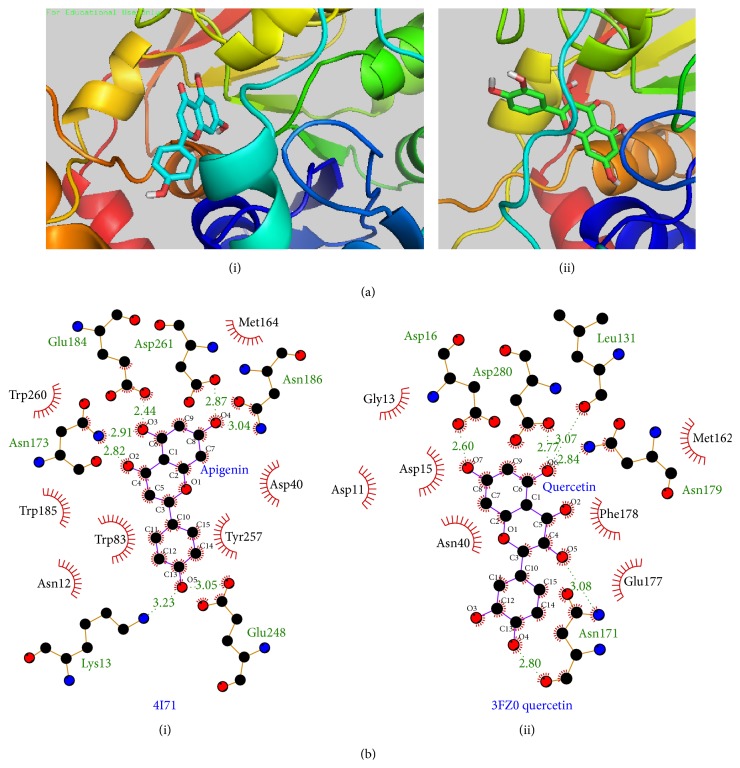
(a) Docked position of (i) apigenin (stick figure) in the binding pocket of IAG-NH and (ii) quercetin (stick figure) in the binding pocket of IG-NH. (b) LigPlot figures of (i) apigenin docked in IAG-NH and (ii) quercetin docked in IG-NH. Amino acid residues forming hydrophobic interactions were highlighted in red circles. Amino acids contributing to hydrogen bonds were labelled green and hydrogen bonds were indicated as dotted lines with bond length labelled in green.

Overall, all flavonoids showed higher affinity towards IAG-NH than IG-NH. This could be due to the fact that the IAG-NH structure used for docking has a closed and narrower binding pocket compared to that of IG-NH. Thus ligands that are more planar and sufficiently small are able to fit into the IAG-NH pocket. Upon visualizing, the ligands were closer to the amino acid residues in IAG-NH binding pocket; thus the calculated intermolecular forces appeared to be higher. However, this selectivity towards IAG-NH could be further investigated by docking studies with flexible binding pocket or molecular dynamics.  Figure 8 shows the docked position of the compounds of the highest binding energy for both enzymes.

It is difficult to directly correlate our results to IC_50_ of the flavonoids proposed by Tasdemir et al. [23]. This is because the control used in the experimental study melarsoprol is a known inhibitor of trypanothione reductase and all the reported IC_50_ are compared with that target and our target enzymes being different.

Tasdemir et al. had concluded that there was no specific trend observed between the chemical structures and antitrypanosomal activities [23]. Several other* in vitro* studies have also reported excellent trypanocidal activities of methylated and glycosylated flavonoid derivatives [23, 24, 33]. A possible explanation to these observations is that the trypanocidal activity of flavonoids may involve multitarget inhibition; thus structure-activity relationship of flavonoids differs for each protein target. This idea is supported by the fact that flavonoids possess a wide range of bioactivities and are able to inhibit a large variety of enzymes which spans across almost all enzyme classes including hydrolases, oxidoreductases, isomerases, kinases, and ligases [17, 19, 22].

From our results, we showed that the purine salvage pathway enzymes could also be one of the targets. Further docking studies by screening a library of flavonoids for activities against other target enzymes of trypanosomiasis are an ongoing research of our group. Laboratory experiments using specific enzymes could be used to further confirm the inhibition effect of flavonoids on each enzyme. As the laboratory data is still scarce, a more cost-effective alternative is to utilize molecular dynamics simulation for prediction of the protein-ligand interactions.

## 4. Conclusion

Using computational docking studies we have proposed that nucleoside hydrolases could be one of the trypanocidal targets of the flavonoids proposed by Tasdemir et al. in their experimental work. Using several flavonoids structures possessing hydroxyl groups at various positions we have tried to provide insight into the effect of hydroxylation on the free binding energy of the tested compounds. Based on our results flavonoids have more affinity for IAG-NH as compared with IG-NH. The size of the binding pocket and the lining residues favor planar hydrophobic residues with three hydroxyl groups at 5, 7, and 4′ positions of the flavone structure. Further studies using molecular dynamics may provide better insight into the lead flavonoid structure for experimental work.

## Figures and Tables

**Table 1:** Test compounds and their hydroxylations.

Test compounds	Position of -OH
Flavone	—
5-Hydroxyflavone	5
7-Hydroxyflavone	7
Chrysin	5,7
Apigenin	5,7,4′
Kaempferol	3,5,7,4′
Fisetin	3,7,3′,4′
Quercetin	3,5,7,3′,4′

**Table 2:** The test compounds, chemical group classification, and PubChem identification numbers.

Compound	Class	PubChem ID
AGV^a^	Iminoribitol	25141279
BTB^b^	Tertiary amine	81462
Guanosine	Purine nucleoside	6802
Inosine	Purine nucleoside	6021

Flavone	Flavone	10680
5-Hydroxyflavone	Flavone	68112
7-Hydroxyflavone	Flavone	5281894
Chrysin	Flavone	5281607
Apigenin	Flavone	5280443
Kaempferol	Flavan-3-ol	5280863
Fisetin	Flavan-3-ol	5281614
Quercetin	Flavan-3-ol	5280343

^a^AGV is (2R,3R,4S)-1-[(4-amino-5H-pyrrolo[3,2-d]pyrimidin-7-yl)methyl]-2-(hydroxymethyl)pyrrolidine-3,4-diol.

^b^BTB is 2-[bis(2-hydroxyethyl)amino]-2-(hydroxymethyl)propane-1,3-diol.

## Abbreviations

HAT: Human African Trypanosomiasis
WHO: World Health Organization
PSP: Purine salvage pathway
NH: Nucleoside hydrolase
IAG*‑*NH: Inosine-adenosine-guanosine nucleoside hydrolase
IG-NH: Inosine-guanosine nucleoside hydrolase
AGV: (2R,3R,4S)-1-[(4-Amino-5H-pyrrolo[3,2-d]pyrimidin-7-yl) methyl]-2-(hydroxymethyl)pyrrolidine-3,4-diol
BTB: 2-[Bis(2-hydroxyethyl) amino]-2-(hydroxymethyl)propane-1,3-diol.
